# Periprocedural complications of cardiac implantable electronic device implantation in very elderly patients with cognitive impairment: A prospective study

**DOI:** 10.1097/MD.0000000000027837

**Published:** 2021-11-19

**Authors:** Fu Guan, Jianjun Peng, Shu Hou, Lihui Ren, Yunan Yue, Guangping Li

**Affiliations:** a Department of Cardiology, Capital Medical University affiliated Beijing Shijitan Hospital, Beijing, China; b Department of Neuropsychiatry, Capital Medical University Affiliated Beijing Shijitan Hospital, Beijing, China.

**Keywords:** cardiac implantable electronic device, cognitive impairment, complication, elderly

## Abstract

Very elderly people (over 80 years) with cardiac implantable electronic devices (CIEDs) indications often have a higher prevalence of aging comorbidity, among which cognitive impairment is not uncommon. This study aimed to investigate periprocedural complications of CIED implantation among very elderly patients with and without cognitive impairment. One hundred eighty patients ≥80 years of age indicated for CIED implantation were included in our study. During hospitalization, the cognitive evaluation was performed according to the Diagnostic and Statistical Manual of Mental Disorders (fifth edition). According to the cognitive test results, patients were divided into 2 groups (90 patients with normal cognitive function and 90 patients with cognitive impairment). Meanwhile, their physical parameters and laboratory measurements were completed. The procedural data and periprocedural complications were collected from both groups. The association between cognitive impairment and periprocedural complications was analyzed using univariate and multiple logistic regression analyses. During a one-month follow-up, the most frequent periprocedural complications in very elderly patients were pocket hematoma and thrombosis events. Cognitively impaired patients had a higher incidence of complications than normal cognitive patients. Multivariate regression analysis showed that cognitive impairment was positively correlated with periprocedural complications in very elderly patients. Cognitive impairment is associated with increased periprocedural complications of CIED implantation in very elderly patients.

## 1 Introduction

The global population aging is progressing in recent decades. As a result, the prevalence of age-dependent chronic diseases is increasing. According to a survey from the year 2013 to 2014, 23 million people over 80 years of age are living in China, which brought widespread social concern.^[1]^ Among this population, cognitive disorders tend to increase with augmented cardiovascular profiles.^[2]^ Cardiac arrhythmias, especially bradyarrhythmias such as sick sinus syndrome and high degree atrioventricular block have a high prevalence in very elderly patients. Improved therapies for heart disease increased the proportion of very elderly patients requiring pacemaker implantation.^[3]^ Meanwhile, atrial fibrillation (AF) has become the most prevalent arrhythmia in individuals aged over 80 years, and this population has a high incidence of cognitive impairment.^[4,5]^ On one hand, AF increases the risk of cognitive impairment or even dementia as a result of cerebral hypoperfusion and micro thromboembolism, leading to silent cerebral ischemia. On the other hand, the bradyarrhythmias could decline the intellectual status in adults.^[6]^ Thus, many older adults with cognitive impairment have cardiac arrhythmic comorbidities and cardiac implantable electronic device (CIED) indications.^[7]^ In the long run, artificial pacemaker improves their cognitive function.^[8]^

Although CIED could improve cognitive impairment as mentioned above, cognitive impairment may also affect the clinical results of CIED implantation. Concerning surgical operations assisted by general anesthesia, patients with pre-existing cognitive impairment are at greater risk of perioperative complications than those without cognitive impairment.^[9]^ There is limited information on whether cognitive impairment also affects periprocedural outcomes in very elderly patients undergoing CIED implantation assisted by local anesthesia. To raise awareness and minimize the periprocedural complications of CIED implantation in very elderly patients, we aim to investigate whether cognitive impairment is associated with periprocedural complications of CIED implantation in this population. It is necessary to define the very elderly to properly study geriatric diseases since diseases in this population vary according to age. Although there are different ways to classify the age of this population,^[10]^ we attempted to define patients aged 80 years or older as very elderly according to our national context,^[1]^ in order to investigate their clinical outcomes in the following study. This defined age is also in line with the overall composition of the patients in our center since patients aged 80 years or older can be admitted to the geriatric unit of our hospital.

## 2 Methods

### 2.1 Study population

This study was conducted at Beijing Shijitan Hospital Capital Medical University. Clinical data of patients were prospectively collected from our center between March 2015 and January 2020. Data were collected in accordance with the principles of the Declaration of Helsinki. Informed consent was obtained at admission by the Ethics Committee of Capital Medical University Beijing Shijitan Hospital, with the patient's approval of medical information being used for clinical research.

Patients aged ≥80 years were included if they met the following inclusion criteria: there was a clear indication for CIED implantation with the assistance of local anesthesia upon admission; and the patient could complete a cognitive function assessment during the hospital stay after CIED implantation. Exclusion criteria included the following: the patient refused to participate in the study; the patient was unable to complete the cognitive assessment due to severe visual or hearing impairment; the patient had a clear history of psychiatric illness and was receiving psychotropic medication; and the patient had an altered mental status or delirium due to the current illness.

### 2.2 Data collection and CIED implantation

Patients were evaluated for cognitive function during their hospital stay after the CIED implantation. Cognitive impairment was diagnosed by the same neuropsychiatric specialist according to the Diagnostic and Statistical Manual of Mental Disorders (fifth edition). The Montreal Cognitive Assessment (MoCA) was used as a brief and reliable screening tool for cognitive impairment. Using a cutoff score of 26, the MoCA has a sensitivity of 0.89 and a specificity of 0.75 for detecting mild cognitive impairment (MCI).^[11]^ A score below 26 was defined as cognitive impairment and equal to or above 26 as normal cognition.^[12]^ The cognitive impairment group further used the Mini-Mental State Examination to detect dementia with a cutoff value of 26 (21–25 for mild, 15–20 for moderate, 10–14 for severe moderate, and 9 points or lower for severe).^[11]^

Before CIED implantation, clinical information was collected that included a history of cardiovascular disease and the risk factors, including hypertension, diabetes, coronary artery disease, and prevalent geriatric diseases, including chronic obstructive pulmonary disease (COPD), venous thrombosis, and chronic kidney disease (CKD). Clinical examinations were performed, including serum tests for international normalized ratio and NT-pro-B-type natriuretic peptide (BNP), and left ventricular ejection fraction rate as defined by echocardiography. The type of device implanted and combined drug therapy were also collected. Periprocedural complications were followed up for 1 month after CIED implantation. Periprocedural complications were defined as pocket infection or hematoma, lead dislodgement or malfunction requiring reintervention, inappropriate shock in defibrillator patients, thrombotic events including ischemic stroke and thromboembolism, pneumothorax due to venous access preparation, and death during follow-up. To assess whether complications were related to cognitive impairment, the same number of patients without cognitive impairment were used as controls in terms of gender, age, and type of CIED implantation. Information on patients was prospectively collected by our team and reviewed retrospectively.

CIED implantation was performed in the right or left subclavian region under local anesthesia. Patients were administered 1.0 g of cephazolin solution (I/V) 6 hours before the procedure. The left or right subclavian vein was chosen as venous access for all patients in our study. All pacing electrodes were active fixation. Patients participating in the study were implanted with the device by the same operator who has 1 or 2 fellow assistants to minimize operating errors. There was no bridging anticoagulation or discontinuation of antiplatelet agents during the periprocedural period. For patients taking anticoagulants routinely, nonvitamin K antagonists, were discontinued 12 hours before the procedure and reinitiated 24 hours after the procedure. Vitamin-K antagonists were continued if the preoperative international normalized ratio was below 2.5 and started 24 hours after the procedure. After implantation, all patients were prescribed 24-hour bed rest and 2 to 4-hour local compressions. Cephazolin was administered 6 hours after implantation and continued for the next 12 hours with 2.0 g. Patients who required temporary pacing prior to CIED implantation had a temporary pacemaker implanted through the femoral vein, which was removed at the end of the procedure.

### 2.3 Statistical analysis

Quantitative variables were described as median (interquartile range, 25%; 75%) or mean ± SD according to their distribution. Categorical variables were presented as percentages. Events were compared by Chi-square test for each group type. Logistic regression analysis was used to calculate the hazard ratio for the risk of periprocedural complications in the cognitive impairment group. *P* < .05 was for comparison between 2 groups.

## 3 Results

### 3.1 Patients characteristics

In this study, we prospectively enrolled 180 patients aged 80 years or older who met the inclusion criteria for CIED implantation at our center from 2015 to 2020. Based on their cognitive assessment by MoCA score with a cutoff value of 26, 90 patients with MCI were grouped as cognitive impairment and 90 with normal cognitive function as noncognitive impairment. In the cognitive impairment group, 15 patients were diagnosed with mild dementia based on a further Mini-Mental State Examination score (22.9 ± 3.1). The clinical characteristics of the cohort were shown in Table 1.

**Table 1:** Baseline information of patients grouped by cognitive impairment.

Baseline characteristics	Total population (N = 180)	Cognitive impairment (N = 90)	Noncognitive impairment (N = 90)	*P*-value
Age (yr)	85 (80–100)	87 (80–95)	84 (80–100)	.450
Male (N, %)	109 (57)	56 (62)	53 (58)	.109
BMI	25 (23–28)	24 (20–23)	25 (23–29)	.224
NYHA class (N, %)				
I	5 (2)	2 (2)	3 (3)	.201
II	95 (53)	48 (53)	47 (52)	.223
III	59 (33)	30 (33)	29 (32)	.500
IV	21 (12)	10 (11)	11 (12)	.488
LVEF (%)	51.6 ( ± 3.8)	52 ( ± 4.4)	51 ( ± 4.5)	.210
BNP	1426 ( ± 777)	1728 (646–1622)	1273 (637–1210)	<.001
ECG (N, %)				
AF	90 (51)	48 (53)	42 (47)	.001
AVB	43 (24)	21 (23)	22 (24)	.333
SSS	87 (48)	42 (47)	45 (50)	.210
VT	6 (3)	3 (3)	3 (3)	.900
Medications (N, %)				
Diuretics	62 (34)	33 (36)	29 (32)	.202
Beta-blocker	5 (5)	3 (3)	2 (2)	.501
ACEI	113 (21)	46 (62)	67 (63)	.900
Amiodarone	5 (5)	2 (2)	3 (3)	.550
Anticoagulants (N, %)				
Vit-K antagonist	36 (20)	16 (17)	20 (22)	.001
NOAC	38 (21)	25 (27)	13 (14)	.001
INR (N = 6)	1.8 (1.6–1.9)	1.8 (1.7–1.9)	1.7 (1.6–1.8)	.981
Comorbidity (N, %)				
Coronary heart disease	91 (50)	45 (50)	46 (51)	.141
Diabetes mellitus	102 (57)	50 (56)	52 (58)	.223
Hypertension	105 (58)	54 (60)	51 (57)	.092
COPD	21 (12)	11 (12)	10 (11)	.137
Chronic kidney disease	66 (37)	28 (31)	38 (42)	<.001
Subclavian vein thrombosis (N)	2	2	0	<.001

± Standard deviation; range 25th to 75th percentile interquartile range. *P* < .05 for comparison between 2 groups. ACE = angiotensin I converting enzyme, AF = atrial fibrillation, AVB = atrioventricular block, II AVB or III degree AVB with pacing indication, BMI = body mass index, BPN = B-type natriuretic peptide (pg/mL), CIED = cardiac implantable electronic device, COPD = chronic obstructive pulmonary disease, INR = international normalized ratio, LVEF = left ventricular ejection fraction, NOAC = nonvitamin K antagonists, NYHA = New York Heart Association I to IV, PVC = premature ventricular complex, SSS = sick sinus syndrome, VT = ventricular tachycardia.

The median age was 85 (range from 80–100). All patients in the study were of Chinese Han ethnicity and living independently in Beijing and its peripheral region. They were retired with previous employment of civil servants, administrators and managers, and workers, which were similar in both groups. The 2 groups had similar comorbidities and most medications, including coronary heart disease, diabetes, hypertension, and COPD. The 2 groups also had similar BMI, comparable NYHA cardiac function classification and left ventricular ejection fraction. However, patients in the cognitive impairment group had higher serum NT-pro-BNP levels than the noncognitive impairment group (1728 pg/mL vs 1273 pg/mL, *P* = .001). In addition, patients in the cognitive impairment group had less CKD (31% vs 42%, *P* < .001) but more subclavian vein thrombosis (2 cases vs 0) compared to the noncognitive impairment group.

As shown in Table 1, the main indication for CIED implantation in very elderly patients was sick sinus syndrome (48% of the total). Also, the prevalence of AF was high, accounting for 51% of this population (53% in the cognitive impairment group and 47% in the noncognitive impairment group). Since it was required in our center that very elderly patients should stay in the hospital for 7 days after implantation for pocket suture removal, the hospitalization time was longer than in other populations.

### 3.2 Periprocedural complications

During the one-month follow-up, periprocedural complications in both groups were presented in Table 2. More pocket hematomas were found after CIED implantation in the cognitive impairment group compared with the noncognitive impairment group (11% vs 6%, *P* = .001). Among the patients complicated with pocket hematoma, 8 patients in the cognitive impairment group recovered with local compression combined with intermittent discontinuation of anticoagulants, whereas 2 other patients underwent pocket dissection and pacemaker reimplantation. On the other hand, 6 patients in the noncognitive impairment group were well managed without reintervention. Lead dislodgement occurred in 3 patients in the cognitive impairment group and 1 in the noncognitive impairment group (3% vs 1%, *P* < .01). Moreover, thrombosis events occurred in 15 patients in both groups. In the cognitive impairment group, 4 patients (4%) developed acute cerebral infarction. Of these, 1 patient died of comorbid severe pneumonia during hospitalization, and the other 3 patients survived but developed cerebrovascular sequelae. One patient in the noncognitive group had a transient ischemic attack and recovered during follow-up. Of the 2 patients in the cognitive impairment group who died, 1 was due to end-stage renal failure and the other one to ischemic stroke comorbid severe pneumonia, as previously mentioned. Venous thrombosis was found in 7 patients in the cognitive impairment group (7%) and 3 patients in the noncognitive impairment group (3%). One patient in the cognitive group received an inferior vena cava filter combined with oral anticoagulation due to deep vein thrombosis. Other cases in both groups received continuous oral anticoagulation for superficial venous thrombosis. For venous puncture-related pneumothorax, there were 3 cases (3%) in the cognitive impairment group and 1 case (1%) in the noncognitive impairment group, all of which were managed conservatively with no invasive interventions.

**Table 2:** Periprocedural complications.

Perioperative data (median, IQR)	Total population (N = 180)	Cognitive impairment group (N = 90)	Noncognitive impairment group (N = 90)	*P*-value
Complication (N, %)	45 (25)	31 (34)	14 (15)	<.001
Pocket hematoma	16 (8)	10 (11)	6 (6)	.001
Lead dislodgement	8 (4)	5 (5)	3 (1)	<.001
Thrombosis events	15 (8)	11 (12)	4 (4)	<.001
Ischemic stroke	5 (3)	4 (4)	1 (1)	<.001
Venous thrombosis	10 (5)	7 (7)	3 (3)	.001
Pneumothorax	4 (2)	3 (3)	1 (1)	.002
Death	2 (1)	2 (2)	0	<.001

*P* < .05 for comparison between cognitive impairment group and noncognitive impairment group.

Data were stratified according to the presence or absence of cognitive impairment to determine if there was an association between cognitive impairment and increase periprocedural complications of CIED implantation in very elderly patients. The analysis was performed by adjusting for clinical confounders, including NT-pro-BNP level, anticoagulation therapy due to AF, CKD, subclavian vein thrombosis, and temporary pacing prior to CIED implantation. As shown in Table 3, the risk of periprocedural complications was 1.18 (95% CI, 1.08–1.29) times higher in the cognitive impairment group.

**Table 3:** Multivariable models to assess the association between cognitive impairment and incidence of periprocedural complications of CIED implantation in very elderly patients, adjusting for clinical risk factors.

Cognitive impairment group			
Model	Parameter estimate	*P*-value	HR (95% confidence interval)
Crude model	0.299	.018	1.22 (1.14–1.31)
Adjusted for clinical risk factors^∗^	0.270	.027	1.18 (1.08–1.29)

∗ Adjusted for NT-pro-B-type natriuretic peptide level, anticoagulation therapy due to atrial fibrillation, chronic kidney disease, and subclavian vein thrombosis, and the number of temporary pacing before CIED implantation. Cognitive impairment is associated with more periprocedural complications of CIED implantation in very elderly patients. *P* < .05 for significant comparison.

## 4 Discussion

### 4.1 Major findings

Our prospective observational study described the periprocedural complications of CIED implantation experienced by very elderly patients ≥80 years of age at a one-month follow-up. Periprocedural complications found in this population included pocket hematoma, lead dislodgement, venous thrombosis, ischemic stroke, iatrogenic pneumothorax, and death. The incidence of various complications was compared between patients with cognitive impairment and patients with normal cognitive function. The overall complications rate was higher in cognitively impaired patients (34%) than in cognitively normal patients (15%).

Device implantation in very elderly patients is increasing since they achieve the benefits of CIED implantation on symptoms and quality of life.^[13]^ Data from a Dutch registry showed that 32.6% of patients over 80 years of age underwent initial pacemaker implantation.^[14]^ During aging, the heart undergoes cardiomyocyte death and fibrosis due to oxidative injuries.^[15]^ These pathological changes lead to various clinical manifestations due to cardiac conduction disorders, which may be an indication for CIED. Despite clinical studies confirming the positive effect of cardiac pacing on cognitive function and cerebral blood flow of elderly patients,^[8]^ a higher incidence of periprocedural complications of CIED implantation was still observed in our study population of cognitively impaired patients ≥80 years of age. In this study, we sought to assess the impact of cognitive impairment on periprocedural complications in patients >80 years of age at the one-month follow-up after CIED implantation, in combination with coexisting cardiovascular disease and other geriatric comorbidities.

### 4.2 Periprocedural complications in cognitively impaired patients

In our study, the most common complications at the one-month follow-up were pocket hematoma and thrombotic events, both of which were more frequent in patients with cognitive impairment compared to those with normal cognition. Our present incidence of hematoma and thrombotic events was higher than that observed in previous studies.^[16,17]^ The bleeding and clotting events could be related to the widespread application of oral anticoagulants and the patients’ underlying cognitive impairment. Cognitive impairment might also increase the risk of thrombosis or bleeding due to under- or over-dosing of anticoagulants. Among the cognitively impaired patients, 5 admitted to having experienced forgetting or repeating medications during follow-up, although they did not experience complications. However, the discontinuation/re-initiation of anticoagulation therapy and the effect of different drugs and comorbidities, including heart failure and renal insufficiency, could lead to new bleeding or thrombotic events in the periprocedural period.^[18,19]^ To avoid these errors, medications must be strictly controlled in very elderly patients with cognitive impairment. Their families must be fully informed about the periprocedural precautions after discharge. It would be helpful for physicians to prepare strategies with a proven high safety profile, such as the recommendation of oral anticoagulation during pacemaker implantation at high thrombotic risk.^[20]^ In addition, very elderly patients with cognitive impairment might not reasonably cooperate with CIED implantation under local anesthesia and subsequent medical care, or even postoperative bed rest. These might also lead to an increased risk of hematoma or pocket infection. In this regard, leadless intracardiac pacemakers may be an option.^[21]^

In our study population, lead dislodgement was the second most common complication (4%) and more frequent in the cognitively impaired group. Lead dislodgement underwent reintervention as soon as it was diagnosed. It is not usually easy to associate lead dislodgement with a specific cause. Very elderly patients have more comorbidities such as left/right ventricular enlargement, endocardial fibrosis, and tricuspid regurgitation, which increased the risk of improper positioning of the initial pacing lead. Patients with cognitive impairment may have more difficulties in bed rest and postprocedural follow-up. The following points can be taken as precautions: the device should be placed under the pectoralis fascia and secured with sutures to the underlying muscle; sufficient and the appropriate amount of intravascular lead slack should be allowed so that no tension is applied at the tip during deep breathing or excessive arm movement; and it is recommended that the patient's ipsilateral upper extremity be properly immobilized within 24 hours of implantation.

The incidence of pneumothorax was 2% in the total population and was more common in cognitively impaired patients (3%) compared to cognitively normal patients. One patient in the cognitive impairment group had COPD. All these patients were asymptomatic or had mild dyspnea. Pneumothorax was immediately identified at the time of subclavian vein puncture and required termination of the procedure. Conservative management without invasive drainage was effective in all cases. CIED implantation was later performed during the same hospitalization. In the patients of our study, subclavian vein puncture was performed without image support from venography. Whereas, axillary venous access combined with a venography-guided image would be our future practice for this special population.^[22,23]^

In summary, physicians must balance the benefits and risks of CIED implantation and individualize the management of this special population to obtain safe and effective implantation. In addition, our study has several limitations. The sample size was relatively small and the follow-up period was short. And the study was limited to our single center. We did not separate mild dementia from MCI. Therefore, we could not determine the degree of cognitive impairment required to affect the periprocedural complications of CIED implantation in very elderly patients.

## 5 Conclusions

One-month follow-up showed a high incidence of periprocedural complications with CIED implantation in patients ≥80 years of age. Cognitive impairment is associated with an increase in periprocedural complications in this population. Given that most complications are reversible, cognitive function assessment and close follow-up are strongly recommended for these peculiar patients.

## Acknowledgments

Special thanks were given to Kaiying Jia, Hongli Zhang, Xing Gao, and Li Ding for proofreading the patient follow-up information.

## Author contributions

**Conceptualization:** Fu Guan, Shu Hou, Guangping Li.

**Data curation:** Fu Guan, Jianjun Peng, Shu Hou, Lihui Ren, Yunan Yue.

**Formal analysis:** Fu Guan, Jianjun Peng, Yunan Yue.

**Investigation:** Fu Guan, Jianjun Peng, Shu Hou, Lihui Ren.

**Methodology:** Fu Guan, Jianjun Peng, Lihui Ren.

**Project administration:** Fu Guan, Guangping Li.

**Resources:** Jianjun Peng.

**Software:** Yunan Yue.

**Supervision:** Fu Guan, Guangping Li.

**Validation:** Yunan Yue.

**Visualization:** Fu Guan, Yunan Yue.

**Writing – original draft:** Fu Guan.

**Writing – review & editing:** Guangping Li.
